# Some Health Reports—I

**Published:** 1910-09-24

**Authors:** 


### SOME HEALTH REPORTS—I.

Health Department. Report on the Health of the City of Liverpool during 1909. By E. W. Hope, M.D., D.Sc., Medical Officer of Health. Printed by order of the Health Committee. (Liverpool: C. Tinling and Co., Ltd. 1910. Pp. 284. With charts, photographs, maps, and an appendix.)

A preliminary hasty inspection of this elegant and elaborate volume would hardly prepare one for the apology which the Medical Officer, who is responsible for its appearance, sees fit to make for the brief way in which the many important features of the health administration of a large city like Liverpool are dealt with. The excellent and clear charts with which it abounds, the beautiful and well-reproduced photographs which illustrate the text, and the well-printed maps of insanitary areas, new open spaces and buildings for the housing of the working classes, show, quite apart from the text of the book, that the labour and care of the Health Officer, his staff, and the Health Committee have been unstinted in its production. Liverpool has an area of 16,619 acres, or 26 square miles. Its population is estimated at 760,357, with a density of 45.7 persons per acre. For the year under review it had general, zymotic, infantile, and phthisical death-rates of 18.3, 2.4, 143, and 1.3 per thousand respectively. Its birth-rate was 31.0 per thousand, which is considerably above the average of 25.6 for England and Wales. During the year 13,945 deaths and 23,591 births were registered, of which last 12,116 were males and 11,475 females. The number of illegitimate births was 833, or 3.5 per cent. of the total births. Attention is drawn by the Health Officer to the large number of deaths which occurred in public institutions in this city, namely, 4,609 of residents and 779 of non-residents within its boundaries. This proportion is much higher than that of other towns. He explains the fact partly by the esteem in which these places are held, but mainly as the result of the extreme poverty of the inhabitants, which necessitates their removal in case of sickness to public institutions. Moreover, this has been the case for several years. The following are the principal causes of deaths for the year: Zymotic and septic diseases, 1,881; constitutional diseases, 173; tubercular, 1,503; nervous, 1,446; circulatory, 1,113; respiratory, 3,493; digestive, 921; lymphatic, 18; urinary, 422; reproductive, 95; joints, etc., 29; integumentary, 48; dietetic, 14; developmental, 1,896; and diseases of uncertain or variable seat, 728; causes investigated at coroners' inquests, 973; and causes unspecified, 8. Of zymotic diseases deaths were caused by measles, 471; scarlet-fever, 219; diphtheria, 112; membranous croup, 3; whooping-cough, 228; diarrhœa, 514; influenza, 84; typhus, 8; typhoid, 54; and other zymotics, 188. Nine cases of small-pox were notified, but no death occurred from the disease. Under the Public Health (Tuberculosis) Regulations, 1908, 3,783 cases of phthisis were notified. In addition 917 cases were notified voluntarily. Of these 4,700 cases, 3,048 were males and 1,652 females. An elaborate table is given of the occupations of persons treated at home. A further decline in the number of deaths from excessive drinking is a gratifying feature during the year. The Health Officer attributes this in part to the diminution of facilities for obtaining drink in the poorer parts of the city. It must, however, be remembered that alcohol does not always figure on the death-certificate of those whose death it may be primarily or secondarily responsible for. However, the figures as they stand show a reduction as compared with those similarly obtained for previous years. Deaths from cancer were 694, an increase on previous years. The ratio of males to females dying from the disease was 287 : 407, a female preponderance largely due to the greater incidence in this sex of malignant disease of genito-urinary tract. The greater part of the volume deals with the sanitary condition of the city, including the medical inspection of school children. In all the number of cases of communicable skin and eye conditions dealt with was 17,026, made up of ringworm, 2,168; itch, 571; sore eyes, 3,003; sore heads, impetigo, and eczema, 3,860; other skin diseases, 1,820; verminous cases, 2,681; and neglect cases, 2,923. Space will not allow of extensive reference to the work of the sanitary staff in connection with lodging-houses, mortuaries, milk, factories and workshops, the inspection of food supplies, etc.

